# Optimizing Antiseizure Candidate Compounds Using Deuterium Substitution

**DOI:** 10.1021/acscentsci.6c00941

**Published:** 2026-06-12

**Authors:** Amy Cheng, Renae M. Ryan

**Affiliations:** 1 School of Medical Sciences, Faculty of Medicine and Health, 4334 The University of Sydney, Sydney, New South Wales 2006, Australia; 2 School or Biomedical Engineering, Faculty of Engineering, The University of Sydney, Sydney, New South Wales 2006, Australia

## Abstract

*In vivo* and *in vitro* evaluation of second-generation glutamate transport enhancers with improved developability.

## Graphical abstract

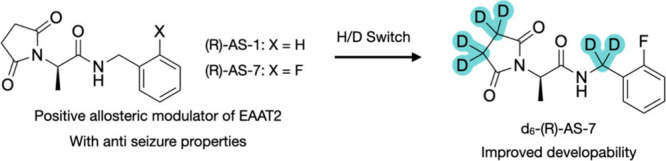

Epilepsy is a complex neurological disorder characterized by recurrent seizures. While several antiseizure medications (ASMs) are in clinical use, over 30% of patients experience drug-resistant epilepsy, defined as achieving no effective response after adequate trial of at least two well-tolerated ASMs.^1,2^ Thus, there is a need to expand the repertoire of drug targets in the development of novel ASMs. In a recent issue of *ACS Central Science*, Kamiński and co-workers utilize a deuterium-switch strategy to improve drug-like properties of the candidate compound (R)-AS-1, which has been shown to display antiseizure activity in mice.^3^ In this study, the authors combine *in vivo* mouse seizure models with *in vitro* characterization to investigate the pharmacokinetics and mechanism of action of a series of deuterated second-generation compounds (Figure 1).

**Figure 1:**
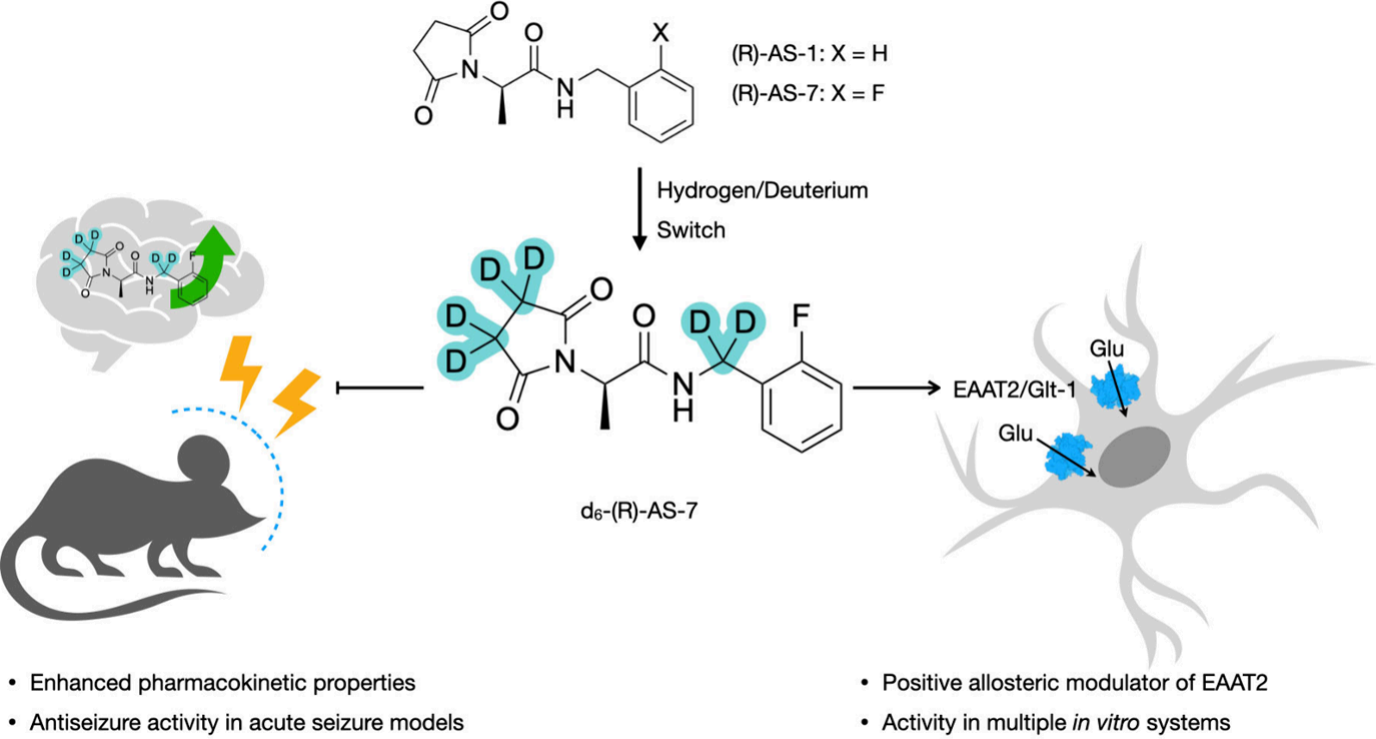
Optimization of lead compounds targeting EAAT2 with antiseizure activity via a hydrogen/deuterium switch strategy.

Kamiński and co-workers utilize a deuterium-switch strategy to improve drug-like properties of the candidate compound (R)-AS-1, which has been shown to display antiseizure activity in mice.

Excitation–inhibition balance in the central nervous system (CNS) is regulated by various ion channels and transporters, the dysfunction of which contributes to hyperexcitability and the pathogenesis of epilepsy.^1^ As such, most current ASMs suppress the occurrence of seizures by inhibiting the glutamate-mediated excitatory systems or activating the GABA-mediated inhibitory systems.^1^ The excitatory neurotransmitter, glutamate, is cleared from the synapse by a family of excitatory amino acid transporters (EAATs), where the EAAT2 subtype facilitates the majority of glutamate uptake.^4^ While the majority of ASM targets are localized in neurons, EAAT2 is predominantly expressed on the plasma membrane of astrocytes surrounding excitatory synapses. Elevating EAAT2 activity, either through upregulation of its expression or via positive allosteric modulators (PAMs) that enhance EAAT2-mediated glutamate clearance, may provide a therapeutic opportunity for epilepsy and other diseases associated with disrupted glutamate homeostasis in the CNS.

In a previous study, Kamiński and colleagues developed a lead compound (R)-AS-1 (Figure 1) that functions as a PAM of EAAT2, which is predicted to bind to a site distal to the glutamate binding site and demonstrates enhanced uptake of radiolabeled glutamate in COS-7 cells expressing EAAT2.^5^ This compound displays favorable antiseizure activity in mouse models but exhibits a relatively short elimination half-life, limiting its therapeutic potential.^5^ Deuteration of small molecule drugs is a strategy that has been used to improve chemical and metabolic stability. A prominent example is deutetrabenazine, an FDA-approved drug used to treat involuntary movements associated with Huntington’s disease and tardive dyskinesia, which exhibits increased elimination half-life and systemic exposure compared to nondeuterated tetrabenazine.^6^ In this study, the pharmacokinetic profile of (R)-AS-1 and its fluorinated counterpart (R)-AS-7 was improved by a rational hydrogen/deuterium switch. The deuterated derivatives reach peak serum concentration within the first hour of intraperitoneal administration in male CD-1 mice comparable to the parent compounds, while most of the deuterated compounds also displayed prolonged systemic and brain exposure and longer elimination half-lives relative to the parent compounds. The authors also note improved brain exposure of the deuterated derivatives of (R)-AS-7; in the case of (R)-AS-7-*d*_6_, this profile was also observed after oral administration.

This study employed multiple acute seizure models to assess the antiseizure activity of the deuterated compounds in male CD-1 mice. In agreement with the improved pharmacokinetic profiles, the deuterated compounds also performed better than the parent compounds at a longer pretreatment time point. The deuterated, fluorine-containing lead compound (R)-AS-7-*d*_6_ displayed higher or comparable potency to clinically relevant ASMs with similar safety profiles in CD-1 mice. Furthermore, (R)-AS-7-*d*_6_ displayed antiseizure activity in a 6 Hz (32 mA) model in both male and female C57BL/6J mice.

In this study, the pharmacokinetic profile of (R)-AS-1 and its fluorinated counterpart (R)-AS-7 was improved by a rational hydrogen/deuterium switch.

Concerns about the effectiveness of earlier PAM’s of EAAT2 have been raised in two separate studies where enhancement of EAAT2 activity by Parawixin 10, isolated from *Parawixia bistriata* spider venom,^7^ or a related small molecule GT949 ^8^ were not able to be replicated. To address these concerns, Kamiński and colleagues employed multiple heterologous expression systems and different astrocyte-based assays to characterize the activity of (R)-AS-7-*d*_6_. Radiolabeled glutamate uptake assays were performed in COS-7 cell lines expressing EAAT2 across different laboratories as well as in mouse and rat astrocyte cultures, demonstrating that (R)-AS-7-*d*_6_ enhanced glutamate uptake with potency in the low nanomolar range. Increased synaptic transporter currents in mouse astrocytes of hippocampal brain slices and glutamate-activated currents in *Xenopus laevis* oocytes expressing EAAT2 were also observed, albeit requiring higher doses of the compound.

Mechanistically, inhibition of excitatory synaptic transmission via enhancing EAAT2 activity represents a new target distinct from current ASMs, but further preclinical evaluation in more advanced and disease-relevant models is required. In addition to clearing glutamate from the synapse, the EAATs also facilitate a chloride conductance, which may contribute to chloride homeostasis in the CNS.^9,10^ Docking of the parent compound (R)-AS-1 to EAAT2 ^5^ indicates it binds near the extracellular region of the chloride conducting pathway,^10^ and functional characterization of patient variants in EAAT1 ^9^ and EAAT2 ^11^ suggests disruption of the chloride conductance may be associated with neurological disease. While the current study demonstrates that the PAM’s increase glutamate-activated currents mediated by EAAT2, whether they affect both the transport and chloride conducting component remains to be determined through more detailed electrophysiological analysis. Furthermore, confirming direct interactions between the lead compounds and EAAT2 through methods such as site-directed mutagenesis and structural analysis is required to identify the binding site and better understand the mechanism of action of transporter stimulation by (R)-AS-7-*d*_6_ and related compounds.

While there remain challenges in developing novel ASMs, this work demonstrates that stimulation of EAAT2 activity is a potential novel route to treat epilepsy and that deuteration is a compelling strategy for lead optimization, where deuterium substitution at selected positions improves developability of the lead molecules without fundamentally altering antiseizure activity or the proposed mechanisms of action.
